# Hijacked scientific journals: a warning to researchers

**DOI:** 10.1590/S1679-45082016CE3632

**Published:** 2016

**Authors:** Thiago Gonçalves dos Santos Martins, Ana Luiza Fontes de Azevedo Costa, Francisco Javier Solano Moncada, Ricardo Vieira Martins

**Affiliations:** 1 Universidade Federal de São Paulo, São Paulo, SP, Brasil.; 2 Hospital das Clínicas, Faculdade de Medicina, Universidade de São Paulo, São Paulo, SP, Brasil.; 3 Universidade de São Paulo, São Paulo, SP, Brasil.; 4 Universidade Federal do Rio de Janeiro, Rio de Janeiro, RJ, Brasil.

Dear editor,

Hijacking of scientific journals consists of websites created by hackers in place of true academic journals for copying their names, addresses, impact factors and international publication numbers in order to steal scientific output and payment of fees charged for publication.^1^

Today there is great pressure on researchers to publish their scientific results. Some journals publish for free and others charge a fee. Some, among those that charge fees, use the fee to ensure faster publication, but give low concern to quality of reviewing itself.^2^

The hijacking of scientific journals began in 2012, and currently more than 20 scientific journals are on this list.^1,3^ These journals are from different countries such as Switzerland and Austria. They are multidisciplinary journals, usually not originally published in English, accept manuscripts from different areas and have impact factors measured by Thomson & Reuters. Most of these journals are available in-print only, and they are not high impact factor journals, which end up convincing more easily the invited author to publish within weeks. In addition, in this fraud the names of the respective actual journal editors are used without their permission.^3^

Victims are usually selected from scientific journal websites that are not peer reviewed and listed in the Thomson & Reuters or Scopus. Hijackers avoid selecting researchers that publish in high quality journals with well-established peer reviews process, because these authors could scan them more easily. Researchers are usually contacted by these false journals by e-mail offering quick publications with considerable impact factor in exchange for publication fees of more than US$500.00.^3^ Once researchers send the article they receive a message with bank information to pay the fee. To further deceive the author, a list of shallow considerations about the article is sent, suggesting superficial corrections to be done before the publication, which will not occur. However, hundreds of researchers have been deceived worldwide.^1^

This is an alarming practice: researchers may have their work stolen and editors the defamation of their journals’ prestige. It is time to think about how much this scientific publication pressure may have made cybercrimes easier. For this reason, we should warn researchers on how to identify hijacked scientific journals.
