# Supplementary material for: Hijacked scientific journals: a warning to researchers
Source: Einstein (Sao Paulo). 2016 Jan-Mar;14(1):113. doi: 10.1590/S1679-45082016CE3632 (PMC4872929; doi:10.1590/S1679-45082016CE3632)
Supplement: Translation

# Translation: Sequestro de periódicos científicos: um alerta aos pesquisadores

**Authors:** Thiago Gonçalves dos Santos Martins, Ana Luiza Fontes de Azevedo Costa, Francisco Javier Solano Moncada, Ricardo Vieira Martins

**Affiliations:** 1 Universidade Federal de São Paulo, São Paulo, SP, Brasil.; 2 Hospital das Clínicas, Faculdade de Medicina, Universidade de São Paulo, São Paulo, SP, Brasil.; 3 Universidade de São Paulo, São Paulo, SP, Brasil.; 4 Universidade Federal do Rio de Janeiro, Rio de Janeiro, RJ, Brasil.

Caro editor,

O sequestro de periódicos científicos é caracterizado por *websites* criados por *hackers* em nome de periódicos acadêmicos reais, que copiam seus títulos, endereços, fatores de impacto e número de publicações internacionais, com o objetivo de roubar a produção científica e os pagamentos feitos aos periódicos para publicação.^1^

Hoje há uma grande pressão para os pesquisadores publicarem seus achados científicos. Alguns periódicos não cobram taxas para publicação, outros sim. Entre os que cobram taxa, há os que o fazem para garantir publicação rápida e sem muita preocupação com a qualidade da revisão do artigo.^2^

O sequestro de periódicos científicos iniciou-se em 2012 e, atualmente, mais de 20 periódicos estão na lista de “sequestrados”.^1,3^ Esses periódicos são de países singulares, como Suíça e Áustria. São periódicos multidisciplinares, geralmente não publicados originalmente em língua inglesa, que oferecem publicações de diferentes áreas e que têm seus fatores de impacto medidos pela *Thomson & Reuters*. A maioria desses periódicos não é eletrônica. Em geral, eles não têm alto fator de impacto, o que convence mais facilmente o autor convidado a publicar em poucas semanas. Além disso, nessa fraude, os nomes dos atuais editores dos periódicos são utilizados sem autorização.^3^

Geralmente, as vítimas são selecionadas diretamente da *homepage* dos periódicos, que não são revisados aos pares e nem listados no *Thomson & Reuters* ou *Scopus*, porém os sequestradores evitam selecionar pesquisadores que publicam em periódicos de alto impacto e com processo de revisão aos pares, já que, obviamente, tais autores podem identificá-los com maior facilidade. Comumente, os pesquisadores são contatados por *e-mail* por esses falsos periódicos, com oferta de publicação rápida e fator de impacto considerável, em troca de taxas de publicação que ultrapassam os US$500.00.^3^ Uma vez enviado o artigo, o pesquisador recebe os dados bancários por *e-mail* para depositar a taxa. Para iludir ainda mais o autor, uma lista de considerações superficiais sobre o artigo é enviada, sugerindo correções mínimas antes da publicação, que, por sua vez, não ocorrerá. Apesar disso, centenas de pesquisadores têm sido enganados em todo o mundo.^1^

Trata-se uma prática alarmante: os pesquisadores podem ter seus trabalhos roubados, e os editores, o prestígio de seus periódicos manchado. É momento de refletir sobre como a pressão pela publicação científica tem facilitado os crimes cibernéticos de ocorrerem. Portanto, devemos alertar nossos pesquisadores de como identificar periódicos científicos sequestrados.
